# Cyclophosphamide-Induced Cardiomyopathy: A Case Report, Review, and Recommendations for Management

**DOI:** 10.1177/2324709613480346

**Published:** 2013-01-01

**Authors:** Sumandeep Dhesi, Michael P. Chu, Gregg Blevins, Ian Paterson, Loree Larratt, Gavin Y. Oudit, Daniel H. Kim

**Affiliations:** 1 University of Alberta, Edmonton, Alberta, Canada

**Keywords:** cyclophosphamide, cardiotoxicity, cardiomyopathy, myocarditis, heart failure, mechanical circulatory support

## Abstract

Cyclophosphamide is increasingly used to treat various types of cancers and autoimmune conditions. Higher doses of this drug may produce significant cardiac toxicity, including fatal hemorrhagic myocarditis. In this review, we present a case of cyclophosphamide-induced cardiomyopathy requiring mechanical circulatory support. We also describe the pathophysiology, clinical manifestations, and risk factors for this important clinical entity and propose early detection and management strategies.

## Introduction

Chemotherapy-induced cardiac dysfunction has become an important cause of morbidity and mortality in patients.^1,2^ The steady introduction of several novel classes of chemotherapeutic agents in combination with off-label use of drugs may also drastically increase the incidence and prevalence of cardiotoxicity.^3,4^ Cyclophosphamide cardiac toxicity is often a lethal complication associated with its use.^1^ Cyclophosphamide is a nitrogen mustard alkylating agent with potent antineoplastic, immunosuppressive, and immunomodulatory properties. Its use has long been established in cancer therapy and in pretransplant stem cell conditioning regimens. Cyclophosphamide’s effect on both cell-mediated and humoral immunity has made it particularly appealing in the off-label treatment of several refractory autoimmune conditions.^5-7^ In light of its variable use, the toxicity profile using distinctive dosing regimens has not been elucidated. Cyclophosphamide-induced hemorrhagic myocarditis has been documented—showing a typical clinical course invariably leading to mortality.^8-11^ The ability to predict cardiac toxicity may allow early and timely interventions, possibly reducing morbidity and mortality.

We describe the first case of cyclophosphamide-induced cardiac toxicity with the successful implementation of mechanical circulatory support in an attempt to bridge to decision, including consideration for cardiac transplantation. We also review relevant literature including pathophysiology, clinical presentation, risk factors for cardiac toxicity, and role of clinical markers for early detection, and we propose potential management strategies.

## Case Presentation

**Figure 1.**
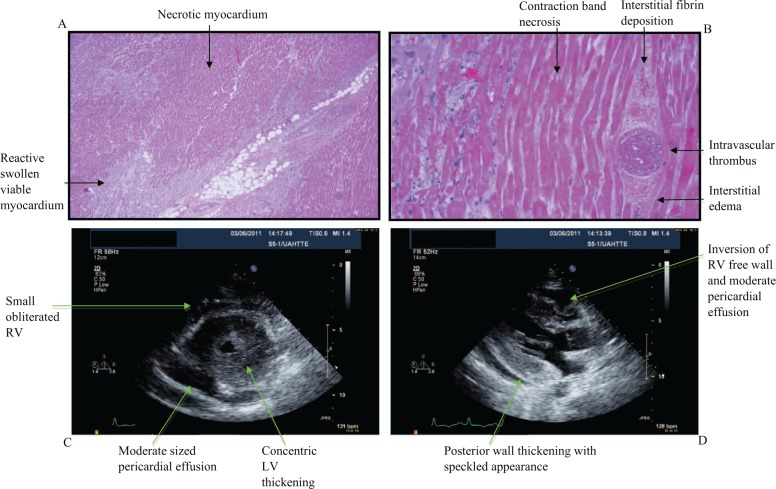
Pathologic and echocardiographic manifestations of cyclophosphamide-induced cardiac toxicity. (A) Hematoxylin and eosin staining of cardiac specimen with areas of necrotic and viable myocardium. (B) Contraction band necrosis, intravascular thrombosis, fibrin deposition, and interstitial edema are seen on high-power field. (C) Basal short axis echocardiographic view showing concentric left ventricular (LV) thickening and a moderate-sized circumferential pericardial effusion. The right ventricle (RV) is small and nearly obliterated. (D) Parasternal long-axis view that shows posterior wall thickening with a speckled appearance. A moderate pericardial effusion is noted with inversion of RV free wall consistent with increased intrapericardial pressures.

A 28-year-old female was initiated on high-dose cyclophosphamide for treatment of refractory neuromyelitis optica spectrum disorder—a group of demyelinating disorders affecting the optic nerve and spinal cord. A baseline, pretreatment multigated acquisition scan demonstrated normal left ventricular ejection fraction of 64%. The patient was started on cyclophosphamide 50 mg/kg intravenously daily for 4 days with mesna protection for hemorrhagic cystitis. By day 4, she became thrombocytopenic and neutropenic prompting the initiation of filgastrim. On day 5, she developed clinical heart failure with rapid deterioration over the next 24 hours. Electrocardiogram (ECG) showed sinus tachycardia with no other abnormalities. Biochemistry showed a troponin I elevation to >30 mmol/L. Urgent echocardiography found severe thickening of the myocardium, a significant pericardial effusion, and evidence of tamponade with right ventricular diastolic collapse and marked variation of right ventricular outflow tract flow during respiration (Figure 1C and D). Despite urgent pericardiocentesis, there was no improvement in her hemodynamic status, and she progressed to pulseless electrical activity. Following initial resuscitation, she was placed on extracorporeal membrane oxygenation. On day 6, signs of neurological recovery were seen and a Levitronix CentriMag (Levitronix, Waltham, MA) biventricular assist device was implanted providing successful mechanical circulatory support as a bridge to decision. Although she remained hemodynamically stable on mechanical circulatory support, her renal function did not recover, requiring continuous renal replacement therapy. The patient went on to develop a large area of ischemic colitis resulting in her death on day 19.

Cardiac autopsy revealed diffuse geographic areas of ischemic necrosis and multifocal thrombosis involving capillaries and larger muscular arterial branches (Figure 1A and B). Frequent contraction bands were seen. The adjacent myocardium showed reactive changes with myocyte swelling, interstitial edema and hemorrhage, and a mixed inflammatory cell infiltrate of macrophages, neutrophils, and lymphocytes. The coronary arteries were free from atherosclerosis and vasculitis, but microvascular thrombosis was evident (Figure 1B). The morphologic findings were compatible with cyclophosphamide-induced toxic myocarditis.

## Pathophysiology of Cyclophosphamide-Induced Cardiomyopathy

**Figure 2.**
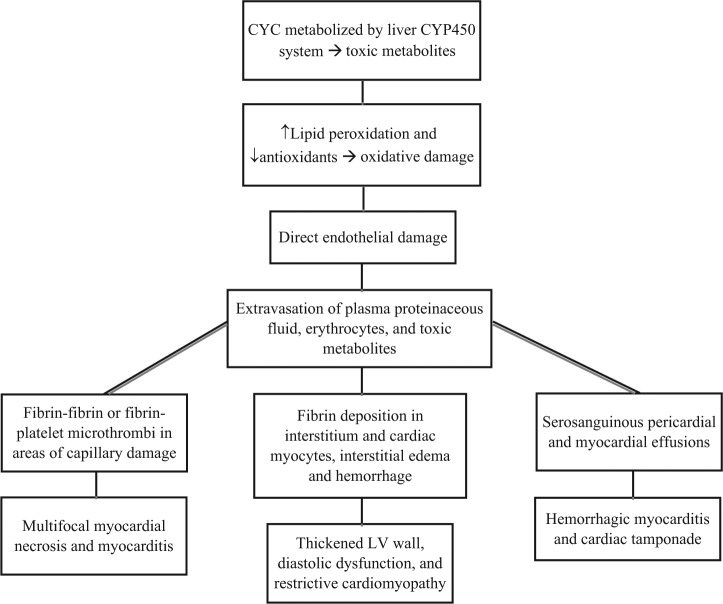
Pathophysiology and clinical manifestations of cyclophosphamide-induced cardiac toxicity. (A) Carboxyphosphamide is an inactive metabolite. (B) Acrolein is associated with hemorrhagic cystitis. (C) Phosphamide mustard is the active cytotoxic metabolite. Abbreviation: LV, left ventricular.

The precise mechanism of cyclophosphamide-induced cardiac toxicity has not been established. Cyclophosphamide metabolites are believed to cause oxidative stress and direct endothelial capillary damage with resultant extravasation of proteins, erythrocytes, and toxic metabolites (Figure 2). Breakdown of endothelial cells in the presence of toxic metabolites causes direct damage to the myocardium and capillary blood vessels resulting in edema, interstitial hemorrhage, and formation of microthrombi. These insults manifest clinically as acute heart failure and arrhythmias. In 1976, Appelbaum et al established the histopathological correlate in 3 out of 4 patients who developed fatal cyclophosphamide-induced cardiotoxicity.^9^ Autopsy results demonstrated increased cardiac weight, intramyocardial extravasation of blood, fibrin or fibrin–platelet microthrombi in capillaries, and fibrin in interstitium. Although many of these findings were nonspecific for myocardial damage, capillary microthrombosis and fibrin deposition in myocardial interstitium were unique to cyclophosphamide-induced cardiac injury. Features in keeping with myocardial necrosis on electron microscopy included hypercontraction bands, myofibrillar damage, lysis, intramitochondrial electron dense inclusions, and fibrin deposition in myocyte cytoplasm. Several subsequent gross pathological examinations have confirmed myocardial edema, thickening of the left ventricular wall and interventricular septum, myocardial necrosis, serosanguinous pericardial effusions, and fibrinous pericarditis.^1,9,12-14^

## Clinical Presentation of Cyclophosphamide-Induced Cardiomyopathy

The spectrum of clinical manifestations from cyclophosphamide-induced cardiac toxicity is variable in presentation and in severity (Figure 2). Common manifestations include tachyarrhythmias, hypotension, heart failure, myocarditis, and pericardial disease.^8,15^ These typically present within the first 48 hours of drug administration but may be seen up to 10 days after initiation. Hemorrhagic myocarditis is a rare complication that is uniformly and rapidly fatal. Once established, it invariably progresses from acute heart failure, to pericardial effusion with tamponade, cardiogenic shock, and eventually to death. The incidence of acute heart failure is anywhere between 7% and 33% of patients receiving a total dose of more than 150 mg/kg cyclophosphamide.^16,17^ Fatal cyclophosphamide cardiomyopathy varies between 2% and 17% depending on different dosing regimens and patient populations. It is difficult to establish the true incidence of cyclophosphamide cardiac toxicity as the literature relies on case reports in which the dose of cyclophosphamide administered is variable, and the drug is often administered in the presence of other cardiotoxins.^9,11,18-22^

## Risk Factors for Cyclophosphamide-Induced Cardiomyopathy

The lack of clearly defined predictive variables for cyclophosphamide cardiac toxicity makes it difficult to determine which patients will be at the greatest risk. There have been several case reports and series of young, otherwise healthy patients developing fatal cardiac toxicity.^9,11,18-22^ A thorough review of the literature highlights several potential risk factors that may be considered when planning therapy. The total dose of an individual course of cyclophosphamide therapy is a well-recognized risk factor for cardiac toxicity, but there is no consensus regarding a threshold dose. Due to its clinical efficacy, cyclophosphamide at 200 mg/kg delivered over 1 to 4 days is commonly employed in refractory cases of several autoimmune conditions.^23^ Early studies performed by Santos et al demonstrated considerable cardiac toxicity at doses more than 270 mg/kg over 1 to 4 days.^26^ Goldberg et al established a dose per body surface area of more than or equal to 1.55 g/m^2^ to produce significant cardiac toxicity.^18^ With its increasing use in stem cell conditioning regimens and refractory autoimmune conditions, there have since been several reports demonstrating significant cardiac toxicity in patients receiving total doses of cyclophosphamide as low as 100 mg/kg.^11^ Specific variations in drug targets and drug metabolism may play an important role in the rapid accumulation of toxic metabolites in genetically predisposed individuals. Drug–drug interactions at the hepatic microsomal cytochrome p450 level also influence the metabolism of cyclophosphamide to its active and toxic metabolites.^20^

In 2000, Brockstein et al found that advanced age and the type of malignancy were independent predictors of cyclophosphamide cardiac toxicity.^22^ Based on their analysis, patients with lymphoma as opposed to breast cancer were at higher risk of cyclophosphamide-induced cardiac toxicity. They postulated that lymphoma might permit immune-mediated enhancement of cyclophosphamide-induced organ toxicity. Patients with preexisting risk factors for ischemic heart disease, prior or concomitant use of other cardiotoxins such as anthracyclines, and a history of radiation therapy to the mediastinum or left chest wall may have elevated baseline risk for cyclophosphamide cardiac toxicity.^20,22,25^ Prior cumulative dose of doxycycline less than 450 mg/m^2^ does not appear to place patients at elevated risk.^20^ Whether a preexisting ejection fraction of less than 50% confers elevated risk is controversial. There have been several negative case series concluding that a resting ejection fraction less than 50% does not confer an elevated risk of cyclophosphamide-induced cardiac toxicity.^15,20,26^ However, this may be attributed to the fact that enrollment criteria for treatment with high-dose cyclophosphamide usually requires an ejection fraction more than 45%. Although little is known about the incidence of cardiac toxicity in patients with severe baseline left ventricular dysfunction, symptomatic heart failure is a reliable risk factor for predicting cardiac toxicity.^22,24^

## Screening and Management of Cyclophosphamide-Induced Cardiomyopathy

Considering the potential for rapidly progressive lethal cardiac toxicity, early detection of cyclophosphamide-related cardiac insult is of paramount interest. The most commonly employed noninvasive method of monitoring cardiac toxicity from chemotherapeutic agents is echocardiography. The earliest changes from cyclophosphamide damage are those of diastolic dysfunction including change in E/A ratio, intraventricular septum thickness in diastole, increased left ventricular diastolic/systolic diameter, and early functional mitral regurgitation.^11,15,21^ Systolic parameters including fractional shortening and ejection fraction may at times be reduced and can be an indication of severe left ventricular dysfunction. However, both depend on preload and afterload, which can be variable in these patients. Cyclophosphamide-induced hemorrhagic myocarditis is associated with hypertrophy, increased myocardial echogenicity, a decrease in left ventricular ejection fraction, and a normal chamber size.^19^ The ECG may be useful in predicting acute heart failure. Prolonged corrected QT (QTc) and increased QTc dispersion, the difference between maximum and minimum QTc interval on a 12-lead ECG, are among the earliest changes in acute heart failure from high-dose cyclophosphamide-containing chemotherapy.^21,27,28^ One study demonstrated an odds ratio of 3.7 (95% confidence interval = 1.5-8.5) with QTc dispersion more than or equal to 10 ms.^21^ Some authors believe QTc dispersion and prolonged QTc may be even more effective than echocardiography. Other common nonspecific findings for cyclophosphamide cardiac toxicity on ECG include reduction in QRS voltage and ST-segment or T-wave changes.^8,15,29^ Cardiac magnetic resonance imaging (MRI) is recommended for the diagnosis of myocarditis and is increasingly used in infarction with hemorrhagic transformation.^30,31^ In patients receiving high-dose cyclophosphamide, cardiac MRI has been used to detect early left ventricular remodeling, but it has not been used to characterize tissue changes in myocardium.^32^

**Figure 3.**
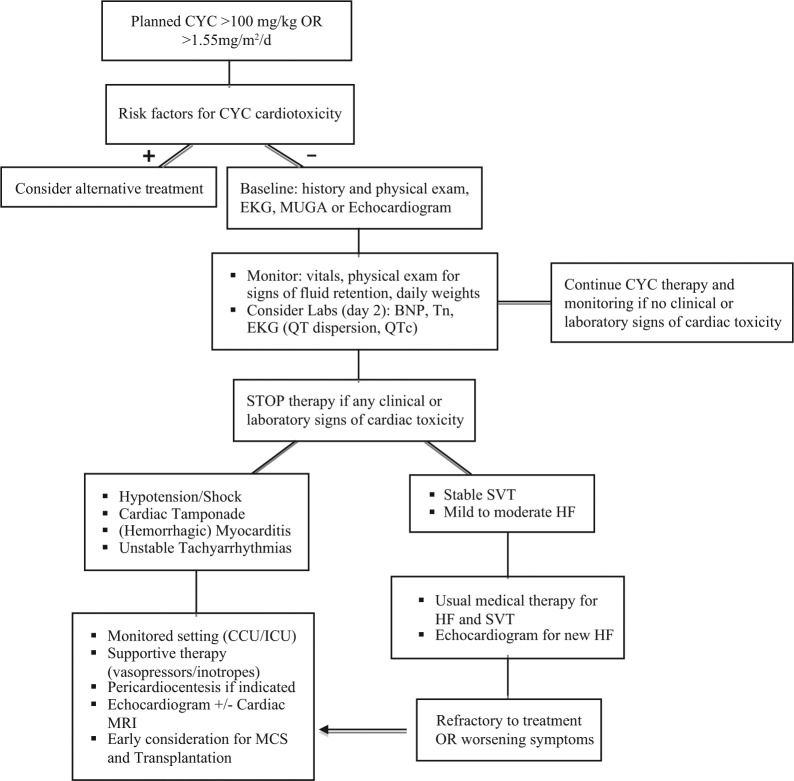
Algorithm for cyclophosphamide initiation and monitoring for cyclophosphamide cardiac toxicity. Abbreviations: CCU, coronary care unit; ICU, intensive care unit; MCS, mechanical circulatory support; MUGA, multigated acquisition scan; SVT, supraventricular tachycardia.

Circulatory cardiac markers may also be valuable in predicting chemotherapy-induced early cardiac toxicity. B-type natriuretic peptide (BNP) is perhaps the most promising in the setting of high-dose cyclophosphamide, since it is elevated within the first 24 hours of therapy and remains persistently elevated for up to 1 week after the clinical presentation of acute heart failure.^33-35^ Cyclophosphamide causes diastolic dysfunction through direct endothelial damage and release of proteinaceous material into the interstitium early in its course (Figure 2). BNP is subsequently released as a response to increased cardiac filling pressures. Monitoring of highly sensitive plasma cardiac troponin I or troponin T, specific markers of myocardial damage, may also have some value in the monitoring of cyclophosphamide-induced cardiotoxicity. Troponin level tends to peak anywhere between 8 and 15 days after the administration of high-dose cyclophosphamide and is generally indicative of direct myocardial damage.^15^ Early troponin elevation in the absence of myocardial damage may be seen after cyclophosphamide administration in the presence of supply–demand mismatch ischemia or in the setting of renal impairment. A general schematic approach to the baseline evaluation and subsequent sttif in the management of patients initiated on cyclophosphamide is provided in Figure 3. Cyclophosphamide therapy should be stopped with any potential clinical or laboratory signs of cardiac toxicity.

Once diagnosed, the treatment of cyclophosphamide-induced heart failure and arrhythmias should be no different from the general approach.^15^ Diuretics, angiotensin-converting enzyme inhibitors, and β-blockers should be instituted early if there are no contraindications. Mild to moderate heart failure and small pericardial effusions generally resolve within a few days to weeks after discontinuation of cyclophosphamide. In 2005, Kamezaki et al published a case report describing severe cyclophosphamide-induced cardiomyopathy that responded to supportive care and additional therapy including theophylline (5 mg/kg/d), a nonselective adenosine antagonist, and ascorbic acid (4 g/d), an antioxidant, with full reversal of the echocardiographic findings at 13 days.^11^ Lee et al had previously described this approach with survival in 1 out of 3 patients with severe acute cardiomyopathy.^12^ The evidence for these therapies remains limited, and the mechanism of action is not well understood. In the presence of suspected cardiac tamponade, hemorrhagic myocarditis, and cardiogenic shock, early recognition and involvement of the intensive care unit or coronary care unit are imperative (Figure 3). Based on our experience, these patients require aggressive monitoring and circulatory support for hemodynamics. Early diagnosis and interventions such as the consideration of extracorporeal membrane oxygenation and mechanical circulatory support to prevent hypoperfusion-related injury and death are required. Due to its almost uniform fatality, the natural history of cyclophosphamide-induced severe cardiomyopathy in the presence of mechanical circulatory support is not known. In the absence of alternative effective therapies, mechanical circulatory support may be a valuable tool for bridge to decision, recovery, or possibly cardiac transplantation.^36-40^ In the setting of severe anthracycline-induced dilated cardiomyopathy, there have been several cases where the use of a mechanical circulatory support allowed for ventricular unloading, recovery of left ventricular function, and reversal of cardiac remodeling within 1 year of implantation.^41,42^ Rapid recognition and initiation of a similar approach in severe cyclophosphamide-induced cardiac toxicity may prove efficacious and may lessen the hypoperfusion-related complications.

## Concluding Remarks

Although we have outlined an algorithm detailing sttif that should be considered when planning cyclophosphamide therapy, the efficacy of this approach has not been validated in the clinical setting (Figure 3). Cyclophosphamide has been used for several decades, but the pathophysiology of cyclophosphamide-induced cardiac toxicity remains poorly understood. The minimum dose for cardiac toxicity is still not known, although there are no reports of cyclophosphamide toxicity at less than 100 mg/kg. Moreover, differences in genetic susceptibilities to this potentially lethal medication have not been evaluated and may explain some of the variability seen in cardiotoxicity based on conventional dosing. The greater off-label use of cyclophosphamide and other chemotherapeutic agents may increase the incidence of chemotherapy-induced cardiotoxicity, and clinicians need to be very aware of the diagnosis and management of this condition. Although the effect of early mechanical circulatory support implementation on overall outcome remains uncertain, it has the potential to support patients with an otherwise rapidly deteriorating course, allowing time for more rational and aggressive interventions, such as cardiac transplantation, which may be the only viable option for this end-stage cardiac toxicity.
